# Functional relations between locomotor performance traits in spiders and implications for evolutionary hypotheses

**DOI:** 10.1186/1756-0500-3-306

**Published:** 2010-11-16

**Authors:** John Prenter, Diana Pérez-Staples, Phillip W Taylor

**Affiliations:** 1 Department of Biological Sciences, Macquarie University, Sydney, NSW 2109, Australia; 2 Instituto de Biotecnología y Ecología Aplicada (INBIOTECA), Universidad Veracruzana, Apartado Postal 250, CP 91090, Xalapa, Veracruz, Mexico

## Abstract

**Background:**

Locomotor performance in ecologically relevant activities is often linked to individual fitness. Recent controversy over evolution of extreme sexual size dimorphism (SSD) in spiders centres on the relationship between size and locomotor capacity in males. Advantages for large males running over horizontal surfaces and small males climbing vertically have been proposed. Models have implicitly treated running and climbing as functionally distinct activities and failed to consider the possibility that they reflect common underlying capacities.

**Findings:**

We examine the relationship between maximum climbing and running performance in males of three spider species. Maximum running and climbing speeds were positively related in two orb-web spiders with high SSD (*Argiope keyserlingi *and *Nephila plumipes*), indicating that for these species assays of running and climbing largely reveal the same underlying capacities. Running and climbing speeds were not related in a jumping spider with low SSD (*Jacksonoides queenslandica*). We found no evidence of a performance trade-off between these activities.

**Conclusions:**

In the web-spiders *A. keyserlingi *and *N. plumipes *good runners were also good climbers. This indicates that climbing and running largely represent a single locomotor performance characteristic in these spiders, but this was not the case for the jumping spider *J. queenslandica*. There was no evidence of a trade-off between maximum running and climbing speeds in these spiders. We highlight the need to establish the relationship between apparently disparate locomotor activities when testing alternative hypotheses that yield predictions about different locomotor activities. Analysis of slopes suggests greater potential for an evolutionary response on performance in the horizontal compared to vertical context in these spiders.

## Background

Locomotor performance of individual animals reflects underlying variation in morphology and physiology and has fitness consequences over a range of ecological and evolutionary contexts [1]. Inferior locomotor performance may have important repercussions in terms of increased risk of predation, reduced intraspecific competitive success and reduced survival [2,3]. Maximum locomotor performance is commonly assessed as burst speed, which may be determined through a variety of experimentally distinct challenges in laboratory and field studies. In spiders, burst speed has been examined in both horizontal running and vertical climbing, and studies have addressed predator avoidance, foraging/predatory behaviour and mate searching ability separately [4-8]. However, the question of to what extent different tests of burst speed assay common underlying capacities has not been examined.

Spiders show the most extreme cases of sexual size dimorphism (SSD) in terrestrial animals, especially some web-building spiders in which females are often massive compared to males [9-11]. Small male size has been repeatedly suggested to promote locomotion and dispersal in males [12-14] and explanations of the evolution of SSD in spiders have explored the link between SSD and locomotor capacity. Recent debate over the role of locomotor performance in the evolution of extreme SSD in spiders proposes speed advantages for small males in climbing in species where males must travel vertically to reach mates [14,15], and alternatively for large males running in ground-dwelling species [5]. These conflicting arguments implicitly treat climbing and running as different performance traits subject to different selection pressures. To date, the validity of this presumption has not been tested empirically.

In general, different assays of locomotor performance and burst speed are examined in isolation. An exception is the study of constraints and evolutionary trade-offs. Evolutionary trade-offs may result from conflicting demands of opposing performance traits [16] that selection cannot maximize simultaneously. There is evidence of such a trade-off between sprinting performances and endurance capacity in different vertebrate groups [17] and sprinting ability and clinging ability in chameleons [18]. Despite the central role of locomotion in evolutionary hypotheses, such trade-offs have not been empirically investigated in spiders.

Here we examine relations between maximum climbing and running burst speed in males of three spider species, two web-builders with high SSD (*Argiope keyserlingi *and *Nephila plumipes*), and a jumping spider (*Jacksonoides queenslandica*) with low SSD. If running and climbing represent common locomotor capacities, then we predict a positive relation between performance ability in each. If they are different capacities with different morphological, physical and physiological demands, then we do not expect a relationship between them. If, however, the physiological and morphological demands of running and climbing in spiders oppose, we expect a trade-off that would be evident as a negative relation between running and climbing ability. We also examine the influence of size on the relation between running and climbing performance because the existence and direction of size-dependent locomotor performance ability in spiders has been the subject of much debate [4-6,14,15]. Furthermore, size is known to influence whole-organism locomotor performance in diverse taxa [2]. As our ability to detect any functional relationship between running and climbing performance relies on a suitably high degree of repeatability in individual performance [17], we also investigate the repeatability of maximum running and climbing performance.

## Methods

### Locomotor assays

We measured the maximum climbing speed and maximum running speed of 35 male *A. keyserlingi*, 25 male *N. plumipes *and 31 male *J. queenslandica*. As climbing speed is influenced by substrate diameter [6], climbing performance was examined on substrates of 0.6, 1.6 and 2.5 cm diameter. We measured cephalothorax width and length for each spider (see Additional file 1).

### Statistical Analysis

Because we were interested in maximum performance, we used only the fastest of three trials in each assay in statistical analyses. This approach mitigates variance due to low motivation and sub-maximal performance [19]. We examined the relationship between maximum climbing and running performance in spiders using random coefficients models in SAS (v. 9.1), with climbing speed as the dependent variable and running speed as the predictor variable. This estimates individual slopes for spiders and then estimates the overall slope for the population [20]. We assumed an unstructured covariance matrix for the intercept and slope. Non-significant quadratic terms and two-way interactions between dowel diameter and running speed were removed from models. As size is known to effect running speed in *J. queenslandica *[6] and is central to motility explanations of SSD in spiders, we also included it in the model. We derived a measure of fixed body size from the factor scores of a principal components analysis of cephalothorax width and length. As the slope of the relationship between running and climbing speeds might affect the potential for selection on each, we also investigated whether the observed slope differed significantly from a 1:1 relationship. Performance data were natural log transformed prior to analyses.

Repeatability (*R*), variation in performance resulting from inter-individual differences, is estimated by the intraclass correlation coefficient (*I_cc_*) [21]. We calculated the intraclass correlation coefficient between the fastest and the next fastest trails for each assay of locomotor performance to determine repeatability of running and climbing performance [5,6] using SPSS 16.

## Results

Maximum running and climbing speed varied across species (Table 1), with males of the orb-weaver *A. keyserlingi *running and climbing faster than the other species. The jumping spider *J. queenslandica *climbed faster than the orb-builder *N. plumipes*, but ran slower. Individual climbing and running performance was generally highly repeatable (Table 1).

**Table 1:** Locomotor performance in spiders

		speed (cm/s)			
	n	climbing			running
		0.6 cm	1.6 cm	2.5 cm	
*A. keyserlingi*	35	9.36 ± 0.62 [0.545]	7.47 ± 0.57 [0.645]	6.25 ± 0.46 [0.503]	33.37 ± 2.45 [0.614]
*N. plumipes*	25	4.72 ± 0.33 [0.867]	4.97 ± 0.51 [0.710]	4.19 ± 0.40 [0.891]	4.70 ± 0.30 [0.730]
*J. queenslandica*	31	5.68 ± 0.30 [0.482]	5.60 ± 0.40 [0.815]	5.91 ± 0.46 [0.735]	3.98 ± 0.45 [0.553]

Maximal climbing and running speeds in male spiders (means ± s.e.). Repeatability (*R*) of performance (intraclass correlation coefficients, *I_CC_*) is given in parentheses.

**Figure 1:**
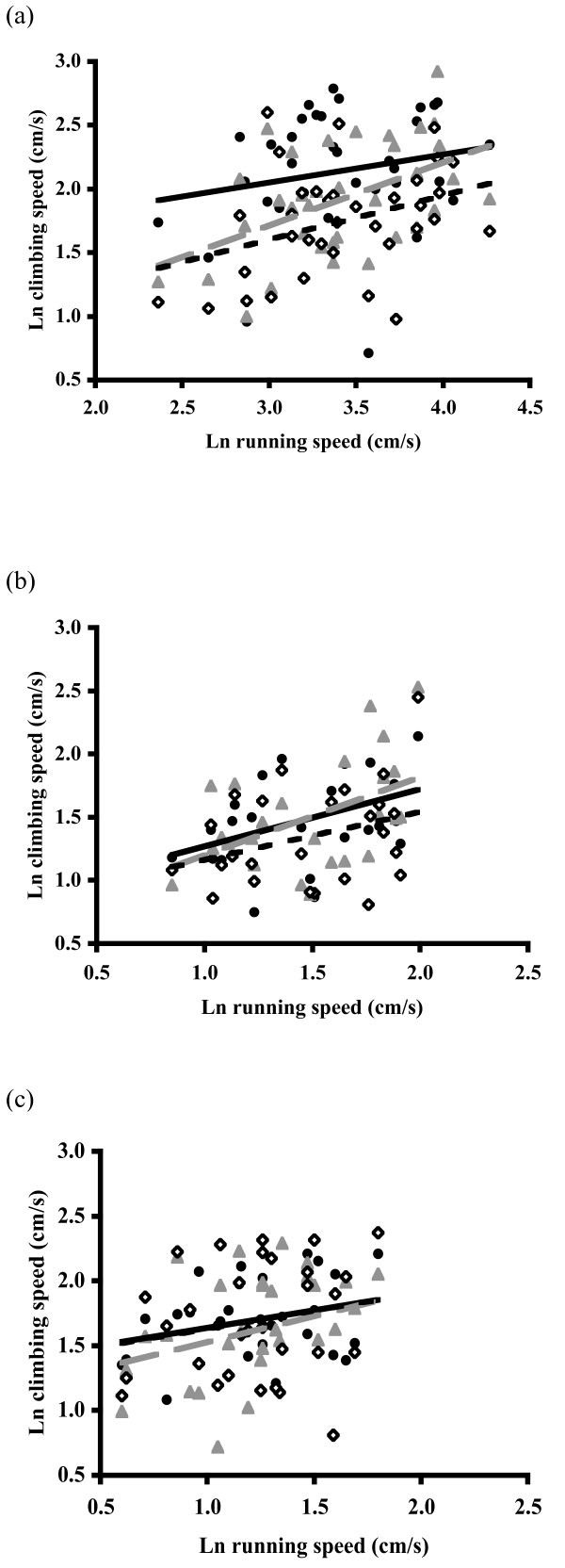
**Inter-relation between two locomotor performance traits in orb-web and jumping spiders**. Positive relation between running and climbing speeds observed in males of (a) *A*. *keyserlingi*, and (b) *N. plumipes*, but not (c) *J. queenslandica*. Climbing was assessed on (1) 0.6 cm (black filled circles, solid line), (2) 1.6 cm (grey filled triangles, broken line), and (3) 2.5 cm (open diamonds, black dotted line) diameter dowels [see Additional File 1].

As is expected if running and climbing represent common locomotor capacities, maximum climbing speed increased with maximum running speed in the highly size dimorphic orb-weavers *A. keyserlingi *(AIC = 116.8, F_1,35 _= 8.48, P = 0.006, β = 0.386, Figure 1a) and *N. plumipes *(AIC = 41.0, F_1,25 _= 7.15, P = 0.013, β = 0.512, Figure 1b). The slopes of both these positive relationships differed significantly from a 1:1 slope (*A*. *keyserlingi*: *t*_33 _= 4.63, P < 0.0001; *N. plumipes*: *t*_23 _= 2.47, P = 0.020). No relation was found between maximum running and climbing speed in the jumping spider *J*. *queenslandica *(AIC = 88.2, F_1,31 _= 0.28, P = 0.600, β = 0.174, Figure 1c; comparison to 1:1 slope, *t*_29 _= 7.09, P < 0.0001). Substrate diameter was negatively related to climbing speed in *A. keyserlingi *(F_1,34 _= 22.86, P < 0.0001, β = -0.021) and *N. plumipes *(F_1,24 _= 6.48, P = 0.018, β = -0.007) but not in *J. queenslandica *(F_1,30 _= 0.03, P = 0.869, β = -0.001). Consistent with Prenter *et al*. [6], body size affected climbing speed only in *J*. *queenslandica *(F_1,31 _= 4.34, P = 0.046, β = 0.113).

## Discussion

In the web-building spiders *A. keyserlingi *and *N. plumipes*, males that were fast runners were also fast climbers. This suggests that climbing and running largely equate to a single locomotor performance characteristic in these spiders. As variation in performance is considered to be rooted in morphological and physiological specialization in animals, high performance in running and climbing may be promoted by the same morphological and physiological characteristics in these spiders. In contrast, the absence of a relation between maximal climbing and running speeds in the jumping spider *J. queenslandica *suggests that we assessed performance in two different (but non-conflicting) modes of locomotion. We would predict, therefore, differences in the morphological and physiological characteristics that promote high performance in these distinctive locomotor modalities. Data for all three species are consistent in the absence of evidence for a trade-off between running and climbing performance. We found no evidence of a negative relation between maximum running and climbing speeds in any of the spiders examined that would indicate a trade-off between running and climbing ability.

While there is no support for trade-offs between running and climbing, it is likely that trade-offs exist between other performance measures. A trade-off between sprinting and endurance capacity is known in various animal taxa [16,17,22], but whether it exists in spiders remains to be determined. Indeed foraging mode may influence selection for these two putatively antagonistic performance traits, with sprinting paramount in sit-and-wait foragers and adaptation for endurance being important in active foragers [8]. The trade-off between sprinting and endurance capacity is normally considered to be influenced by different muscle fibre types that are associated with endurance and sprinting performance [16]. However, spiders differ from the groups so far examined in that hydrostatic pressure plays a key role in their leg movements during locomotion [23]. It would, therefore, be intriguing to determine whether such a trade-off exists in the hydrostatically assisted movement of spiders. There remains potential for different muscle fibres to influence sprinting and endurance capacity in spiders, as the hydrostatic pump controlling this system has been determined to lie in the muscles of the cephalothorax and leg flexion is still achieved using leg muscles [24,25].

Intra-individual variation and low number of trails per individual are known to result in underestimation of maximal speed and repeatability estimates in locomotor performance trials [26]. However, the standard practice [5,6] of calculating the intraclass correlation coefficient using the fastest and second fastest trials is biased in the opposite direction and compensates for any influence of low number of trials per individual spider. The generally high repeatability in both climbing and running performance indicates that individual differences in performance are stable over trials and is consistent with previous studies of locomotor performance in web and wandering spiders [5-7], and other animals [17,26]. This repeatability of running and climbing performance underlines the reliability of our protocols and establishes a platform for future investigation of mechanisms underlying performance variation and heritability in these locomotor traits [21]. Furthermore, our ability to detect a functional relationship between these performance traits depends on high repeatability of individual performance [17]. The repeatability of a trait also determines the upper limit on heritability and represents an estimate of its potential to react to directional selection [[27], but see [28]]. Without repeatability in performance, selection is unlikely to distinguish between high and low performance levels and there would be no directional selection on locomotor performance.

Our results have implications for motility-based explanations for the evolution of SSD in spiders. Recent debate has focused on two alternative explanations; (1) the 'gravity hypothesis' [14,15] that explains the evolution of extreme SSD in spiders because smaller males have a mating or survival advantage through their ability to climb faster, and (2) that larger males have an advantage in running faster [5]. Empirical tests of the two models concentrate on separate laboratory-based assays of running and climbing performance [4-6,15] (but see [29,30] for field studies), with the implicit (but untested until now) assumption that climbing and running represent distinct locomotor modalities. The positive relation between climbing and running performance in male web-spiders in our study contradicts this assumption. Our results highlight the need to consider the relationship between running and climbing performance before embarking on tests that aim to distinguish between alternative motility-based explanations of SSD. In practice it may be difficult to differentiate selection for climbing and running in species where they represent aspects of the same locomotor modality. The direction of the relationship between size and locomotor performance, however, may still retain important discriminatory power. The gravity hypothesis [4,15] predicts a negative relationship between size and climbing speed, while Brandt and Andrade [5] predict a positive relationship between size and running performance. Given the non-independence of running and climbing assays for orb-weaving spiders, selection favouring small males for climbing might also generate a small male advantage in running, and selection favouring large males for running might also generate a large male advantage in climbing.

The slopes of the observed relationships between maximum running and climbing performance were less than unity and suggest that selection cannot act equally on performance on vertical and horizontal surfaces. Our results suggest that the potential for selection on climbing performance (on vertical surfaces) is less than that for running on horizontal surfaces (ground). Any response from selection on body size, via performance, therefore, may differ in the two contexts, with greater potential for a response in the running (ground) context.

## Conclusions

Running and climbing performance were found to be non-independent in the two orb-web spiders *A. keyserlingi *and *N. plumipes*. Assays of these performance traits appear to equate to a single locomotor performance characteristic in these spiders and suggest that high performance in each may be promoted by the same morphological and physiological characteristics. This was found not to be the case for the jumping spider *J. queenslandica*. Our results have implications for motility-based explanations of the evolution of SSD in spiders and suggest greater potential for an evolutionary response in horizontal compared to vertical performance. A trade-off between running and climbing performance in these spiders was not evident.

## Competing interests

The authors declare that they have no competing interests.

## Authors' contributions

JP, PWT and DP-S conceived and designed the experiments. JP and DP-S conducted the experiments. JP and PWT analysed the data and wrote the manuscript. All authors read and approved the manuscript.

## Supplementary Material

Additional file 1 **Additional material**. Click here for file
