# Supplementary material for: Functional relations between locomotor performance traits in spiders and implications for evolutionary hypotheses
Source: BMC Res Notes. 2010 Nov 16;3:306. doi: 10.1186/1756-0500-3-306 (PMC2998518; doi:10.1186/1756-0500-3-306)
Supplement: Additional file 1 — Additional material. [file 1756-0500-3-306-S1.PDF]

Additional material

Additional file 1

Supplemental material and methods

Detailed description of methods relating to collection sites, laboratory maintenance of specimens, morphological measurement and assays of locomotor performance.

Collection and husbandry of spiders

Spiders were collected in Australia as adult males from parks in North Ryde (*A. keyserlingi*), West Pymble, NSW (*N. plumipes*), and from sites around Cairns, QLD (*J. queenslandica*). They were fed 3-4 Queensland fruit flies (*Bactrocera tryoni*) and house flies (*Musca domestica*) on alternate weeks, provided with a constant supply of water and maintained under controlled temperature (24°-26ºC) and relative humidity (65-75%) in individual plastic cages (1L), with a 12:12 hour light:dark cycle.

Morphological measurements

We measured cephalothorax width (mm) and length (mm) of each spider (Table 2) using ImageJ (v. 1.30, National Institutes of Health, Bethesda, MD, USA), from digital photographs (Jenoptik L O S GmbH, Jena, Germany) taken through an Olympus SZX12 stereomicroscope (Olympus Corporation, Tokyo, Japan). To obtain photographs, live spiders were restrained on an upturned 6 cm diameter petri dish using clear plastic film (Glad Products, Padstow, NSW, Australia).

Assays of locomotor performance

We induced spiders to climb three times (with 2 minutes between trials and two hours [see 31, 32] between assays) up a 25 cm dowel by touching the male on its hind legs with a soft-haired brush. In an earlier study [6], substrate diameter affected climbing performance, so we measured climbing speed over the same range of substrate diameters used previously (0.6, 1.6 and 2.5 cm). Maximum running speed was assessed in horizontal raceways (70 cm long x 5 cm wide x 5 cm high) constructed from foam board with a base of fine sandpaper (240 grit) for traction, in three runs [with 2 minutes between runs] over a distance of 60 cm in *A. keyserlingi* and *J. queenslandica*, and 25 cm in *N. plumipes* [6].

References

31. Prestwich KN: **The roles or aerobic and anaerobic metabolism in active spiders.** *Physiol Zool* 1983, **56:**122-132

32. Anderson JF, Prestwich KN: **The physiology of exercise at and above maximal aerobic capacity in a theraphosid (tarantula) spider, *Brachypelma smithi* (F. O. Pickard-Cambridge).** *J Comp Physiol B* 1985, **155:**529-539

Table 2 - Body size and size range of spiders

		carapace width (mm)		carapace length (mm)	
	n	mean	min-max	mean	min-max
A. keyserlingi	35	2.49±0.04	1.70-2.78	2.25±0.04	1.49-2.61
N. plumipes	25	2.20±0.04	1.80-2.55	2.37±0.05	1.80-2.86
J. queenslandica	31	2.30±0.04	1.67-2.64	2.48±0.04	2.06-2.95

Mean (± s.e.) and range (minimum-maximum) of carapace width and length for male spiders used in performance trials. Factor scores derived from a Principal Components Analysis of carapace width and carapace length were used as a measure of fixed body size in statistical models.
